# Gastric emptying times of high-fat, high-carbohydrate and high-protein standard meals using MRI examinations – a contribution to estimating the time since death

**DOI:** 10.1007/s00414-026-03836-8

**Published:** 2026-05-19

**Authors:** Dena Farivar, Birte Hellwig, Anna Holzer, Marcel Alexander Drews, Benedikt Michael Schaarschmidt, Benno Hartung

**Affiliations:** 1 Institute of Legal Medicine, University Hospital Essen, Hufelandstraße 55, Essen, 45147 Germany; 2 Institute for Medical Informatics, Biometry and Epidemiology, University Hospital Essen, Essen, Germany; 3 Institute for Diagnostic and Interventional Radiology and Neuroradiology, University Hospital Essen, Essen, Germany

## Abstract

**Background:**

To use gastric content analysis to estimate the postmortem interval, the last meal consumed, and its gastric emptying time must be known.

However, previous studies have often investigated single foods, liquid meals, or applied heterogeneous protocols, limiting forensic applicability.

Thus, this study aims to investigate gastric emptying times of commonly consumed solid mixed meals with different macronutrient compositions using non-invasive MRI-scans to measure gastric volumes.

**Methods:**

Fifteen healthy participants consumed high-fat, high-carbohydrate, and high-protein meals on separate days, with postprandial MRI-scans every two hours for up to eight hours (total of 232 scans). Gastric half-emptying-times (t₅₀) were calculated via linear interpolation.

**Results:**

The high-carbohydrate meal (t₅₀ 132 ± 33 min) emptied significantly faster than the high-fat (t₅₀ 188 ± 41 min, *p* <0.001) and the high-protein (t₅₀ 158 ± 37 min, *p*=0.010) meals. Women showed on average greater t₅₀-values, significant only for the high-protein meal (*p*=0.003).

**Conclusion:**

This study provides reference values on nutrient-dependent gastric emptying times supporting forensic postmortem interval estimation as a supplementary method.

**Clinical trial number:**

Not applicable.

**Supplementary Information:**

The online version contains supplementary material available at 10.1007/s00414-026-03836-8.

## Introduction

Estimating the time since death is crucial in forensic casework and various methods are used to determine it, such as body cooling, rigor mortis, postmortem lividity, and supravital reactions [1].

Although less common, a forensic scientist must also be prepared to estimate the time since death from gastric contents when such evidence is presented [2]. Previously, estimating the time since death by gastric content analysis has helped to refute witness statements and ultimately solve the case [3, 4].

While qualitative stomach content analyses are performed to identify the last meal of the deceased and its nature (e.g. breakfast, lunch or dinner) [5, 6], or possible intoxications [7], quantitative stomach content analysis aims to estimate the timeframe between the last meal and death [6].

Previous studies have shown that gastric emptying occurs continuously after meal intake and stops at death, as there is no relevant peristalsis to further transport gastric contents into the duodenum [8, 9]. If the last meal consumed by the deceased and the residual stomach content are known, the time interval between meal intake and death can indirectly be estimated by using reference gastric emptying data [10, 11].

Horowitz et al. recommended using only the gastric emptying times of the solid components of a meal since fluids empty from the stomach at different and generally faster rates [11].

However, gastric emptying times vary among individuals and are influenced by various factors such as meal-related factors (e.g. weight, volume, composition, total calorie count) [2], as well as personal factors (e.g. diabetes, pain, stress, increased cerebral pressure) [11].

Current data on gastric emptying times are mostly derived from gastroenterological studies that used standardized test meals (liquids, semi-solids, small portions) to examine isolated effects on gastric emptying [12]. These experimental conditions do not directly reflect everyday meals in which various influencing factors interact.

Tröger et al. provided gastric emptying curves including confidence intervals for mixed meals (containing meat, carbohydrates and plant ingredients) and carbohydrate meals based on autopsy data [10]. Although the authors differentiated between two meal types, there is no description of the initial meal sizes or further details on the included meals. Additionally, the authors compared various gastric emptying studies and drew the following conclusions: a small meal is completely emptied from the stomach after 1–3 h, an average meal after 3–5 h and a large, voluminous meal after 5–8 h. However, the study does not define portion size, nor is it clear if and how meal composition was considered.

Therefore, this exploratory study investigates the effect of different macronutrient compositions on the gastric emptying times of conventionally sized solid meals using magnetic resonance imaging (MRI). It aims to provide reference data that may support stomach content death time estimations in forensic practice.

## Materials and methods

This exploratory study was conducted with prior approval from the ethics committee of the University of Duisburg-Essen (reference number 23-11570-BO). Written informed consent was obtained from all participants before the start of the trial. To obviate the use of ionizing radiation, gastric emptying was assessed with MRI. MRI, which has been validated against the gold standard of scintigraphy [13], allows for repeated non-invasive measurements of gastric content volumes without exposing participants to radiation.

### Subjects

A total of 16 healthy participants were considered eligible for inclusion. Each participant completed a medical history questionnaire to identify potential exclusion criteria in advance. These included conditions that might declare participants unsuitable for MRI-scans or health factors that could influence gastric emptying time (see exclusion criteria, Table 1).

**Table 1:** Inclusion and exclusion criteria for the selection of the study population

Inclusion criteria	Exclusion criteria
• Healthy individuals aged ≥ 18 years	• Pregnancy
• At least 12 h of fasting (no food or fluids) before the beginning of the study	• Pre-existing gastrointestinal diseases (e.g., Crohn’s disease, reflux disease)
	• Conditions affecting gastric motility (e.g., diabetes mellitus)
	• History of abdominal surgery
	• Medications influencing gastrointestinal motility
	• Claustrophobia
	• Known food allergies, intolerances, or vegetarianism, veganism

A total of 241 MRI-scans were carried out between August 2024 and January 2025. 15 participants were initially assumed eligible to participate. However, one participant had to withdraw from the study because of time issues and was later replaced to reach the intended total of 15 healthy participants (6 females and 9 males), who completed all three test days. One participant had to repeat a study day due to the onset of a migraine during the study. The 15 participants were aged between 22 and 29 years (mean 26.1 ± 2.0 years) and mean BMI was 22.55 ± 2.80 kg/m². For characteristics of the study population, see Table 2.

**Table 2:** Characteristics of the study population (*n *= 15)

Characteristics	
Sex	
Female	6 (40%)
Male	9 (60%)
Age (in years)	26.1 ± 2.0
Height (in cm)	177.2 ± 11.4
Weight (in kg)	70.7 ± 9.6
BMI (kg/m²)	22.55 ± 2.80

All values as means ± standard deviation

### Study protocol

Each participant consumed three different meals on three separate days, with gastric filling assessed using MRI.

The three standard meals varied in macronutrient composition (high-protein, high-fat and high-carbohydrate) and were selected based on common dietary patterns:

- Vegetarian rice dish: Chop suey with rice (high-carbohydrate)
- Currywurst (bratwurst topped with curry-flavored ketchup) with French fries and mayonnaise or ketchup (high-fat)
- Half a chicken (roast chicken) with salad and curry ketchup (high-protein)

Meals in this study were considered high of its respective macronutrient, when exceeding the acceptable macronutrient distribution ranges of daily caloric intake [14], which is > 35% of total energy from fats (approx. 9 kcal/g), > 65% from carbohydrates (approx. 4 kcal/g) and > 35% from proteins (approx. 4 kcal/g).

To collect data on the standard meals, three test meals of each meal type were used as „reference meals“, the main components were weighed, and their caloric content and macronutrient composition (carbohydrates, fats, proteins) were calculated using nutritional value tables [15] (What’s In The Foods You Eat *Search Tool*, 2021–2023, Based on the Food and Nutrient Database for Dietary Studies 2021–2023 (FNDDS) [16](Table 3). All meals were ordered from the same restaurants to provide comparable compositions of ingredients and to represent typical portion sizes. Many high-protein diets are rich in fat, too [17]. The selected high-protein meal also meets the criteria for a high-fat meal but is referred to as high-protein due to its major component.

**Table 3:** Meal composition, distribution of macronutrients, energy content, weight, and volume after meal intake of the 3 standard meals

Meal	Composition	Fat (g) kcal (%)	Carbohydrate (g) kcal (%)	Protein (g) kcal (%)	Total energy content (kcal)	Weight (g)	Volume at t0 (ml)
High-fat	Currywurst + french fries + ketchup	69.3 ± 3.0 (51.5%)	122.3 ± 8.5 (40.5%)	24.3 ± 1.5 (8%)	1212.4 ± 29.8	454.0 ± 13.9	718.55 ± 48.1
	Currywurst + french fries + mayonnaise	85.9 ± 3.0 (57.5%)	118.4 ± 8.5 (35,2%)	24.3 ± 1.5 (7.2%)	1344.2 ± 29.8	454.0 ± 13.9	
High-carbohydrate	Chop suey + rice	7.5 ± 1.0 (15.2%)	82,7 ± 4,6 (75.1%)	10.7 ± 0.9 (9.7%)	440.9 ± 30.5	682.3 ± 63.0	835.30 ± 95.9
High-protein	Roast chicken + salad + curry ketchup + dressing	47.5 ± 1.6 (58.9%)	8.4 ± 0.3 (4.6%)	66.0 ± 3.2 (36.4%)	725.7 ± 26.2	602.3 ± 36.0	770.01 ± 99.0

All means were calculated through the examination of 3 test meals for each standard meal ± standard deviation

Kilocalories (%) indicate the proportion of the macronutrient on the total energy content

1 g of fat ≙ 9 kcal, 1 g of carbohydrate ≙ 4 kcal, 1 g of protein ≙ 4 kcal; all volumes at t0 directly after meal consumption are including 200 ml of water, that all participants were required to take in with the meal

Participants consumed the meals on an empty stomach (at least 12 h of fasting; water was allowed up to one hour before the beginning of the study) within 20 min. Each meal was accompanied by 200 ml of water. After the meal, no additional food or liquid intake was planned, although still water was provided ad libitum in 200 ml portions, with consumption recorded accordingly (Supplementary Table 1).

Between the scans, the participants were allowed to sit or walk to mimic the movement of a usual day, but no athletic activity was permitted.

To quantify gastric filling, an initial MRI scan was performed in the fasting state (t-20). Following MRI scans were carried out immediately after meal consumption and then at two-hour intervals, up to a maximum of ten hours after eating. If complete gastric emptying occurred before ten hours had passed, no further scans were performed. Complete gastric emptying was defined as the gastric content volume at the fasting state (t-20) ± 10% of the initial volume at t0, to account for possible water intake during the study.

### MRI technique

All MRI-scans were conducted in the same MRI-scanner (SIEMENS MAGNETOM 1.5T, Siemens Healthineers, Forchheim, Germany) in the Institute for Diagnostic and Interventional Radiology and Neuroradiology, University Hospital Essen, Germany. All scans followed the same protocol of 7 sequences (Table 4). The participants were positioned supine with a radiofrequency coil placed around their abdomen.

Every MRI-scan took about 10 min including intervals of end-inspiratory breath holds to minimize motions artefacts.

The application of contrast medium was not necessary for the MRI examination because the solid meal gave sufficient contrast for volume measurement.

**Table 4:** MR sequence protocol and parameters – gastric region for gastric content determination

Sequence	Orientation	Slice thickness (mm)	Echo Time (TE) (ms)	Repetition Time (TR) (ms)	Field of view (mm)	FoV Phase (%)	Matrix	Flip angle
Localizer	Transversal	6.0	2.45	5.6	430	100	256 × 179	20°
T2_TRUFI_n_cor_6mm	Coronal	6.0	1.85	3.7	380	90.6	256 × 230	54°
T1_Vibe_s_dixon_cor_3mm	Coronal	3.0	(1) 2.39 (2) 4.77	6.7	400	100	288 × 259	10°
T2_HASTE_n_cor_6mm	Coronal	6.0	91.00	1200.0	380	84.4	256 × 256	180°
T1_Vibe_n_dixon_tra_3mm	Transversal	3.0	(1) 2.39 (2) 4.77	6.7	380	81.3	320 × 240	10°
T2_TRUFI_n_tra_6mm	Transversal	6.0	1.85	3.7	380	90.6	256 × 230	54°
T2_HASTE_n_tra_6mm	Transversal	6.0	104.00	1000.0	380	71.9	256 × 205	160°

### Measurement of gastric content volume

**Fig. 1:**
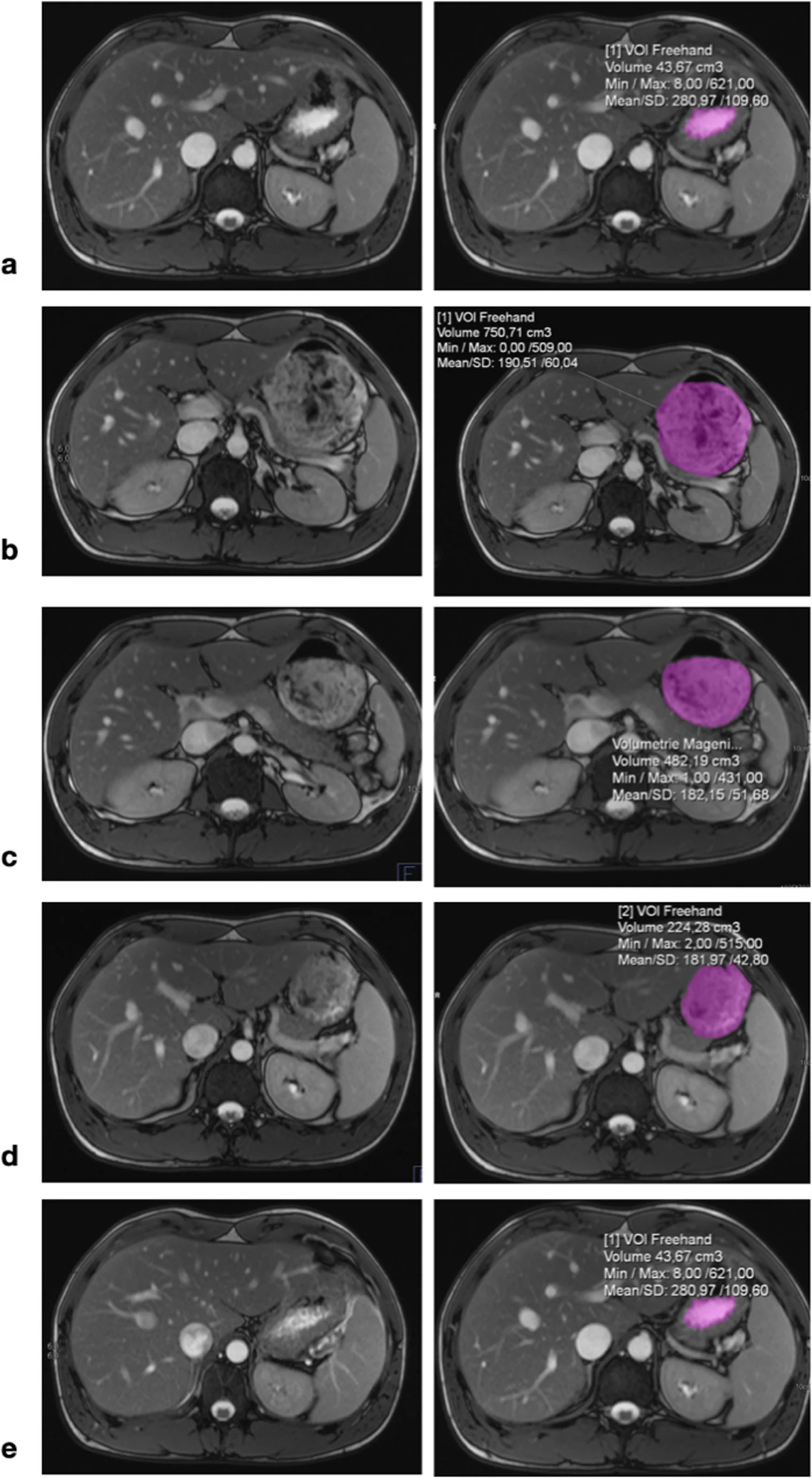
Gastric content assessment by MRI in the axial plane of the TRUFI-sequence. Shown is the gastric content after consumption of the high-fat meal. Even without contrast medium, gastric content shows sufficient contrast for volume determination. MRI-scan (**a**) after 12 h of fasting, (**b**) directly after meal consumption, (**c**) after 120 min, (**d**) after 240 min, (**e**) after 360 min

Gastric content was identified by a clear contrast to the surrounding tissues in the transversal plane of the TRUFI-Sequence. This sequence showed the least motion artefacts while providing sufficient contrast between gastric content and the surrounding tissues and was therefore used for volume determination. Segmentation of the gastric content was performed manually on each slice to calculate the total gastric content volume using syngo.via (Siemens Healthineers, Forchheim, Germany) (Fig. 1). If the gastric area was not completely imaged in the axial plane of the TRUFI-sequence, the coronal plane of the TRUFI-sequence was used for volume determination.

### Data analysis and statistical analysis

Statistical analysis was performed using the software SPSS Statistics Version 29.0 (IBM, Armonk, USA) and Excel (Microsoft 365 Subscription, Microsoft Corporation, USA).

To compare gastric emptying times, gastric half-emptying times (t₅₀) were calculated via linear interpolation and expressed as means with standard deviations. The t_50_-value indicates the time needed for the initial postprandial gastric content volume to decrease by 50%. The Shapiro-Wilk-Test and Q-Q-plots were used to test the data for normal distribution. Possible group differences between meals were assessed using a mixed-design analysis of variance (Mixed ANOVA) with meal type as a within-subjects factor and sex as a between-subjects factor. Significance level was set at α = 0.05. Post-hoc pairwise comparisons within the Mixed ANOVA were Bonferroni-adjusted.

Additionally, t-tests were used to determine possible differences between male and female participants within each meal type. Because of multiple testing, a Bonferroni correction was applied as well, resulting in an adjusted significance threshold of α = 0.017 (0.05/3).

All statistical analyses and results were verified by the Institute for Medical Informatics, Biometry and Epidemiology of the University Hospital Essen (IMIBE University Hospital Essen).

## Results

The high-carbohydrate meal was the biggest meal with an average weight of 682.3 ± 63.0 g and an average volume of 835.30 ± 95.9 ml after meal intake, while the high-fat meal was the meal with the smallest weight and volume (454.0 ± 13.9 g and 718.55 ± 48.1 ml). The high-protein meal sized between (602.3 ± 36.0 g and 770.01 ± 99.0 ml after meal consumption). All volumes measured directly after meal consumption include 200 ml of water that accompanied the meal.

On the other hand, the high-fat meal had the greatest energy content, averaging 1212.4 ± 29.8 kcal when served with ketchup and 1344.2 ± 29.8 kcal when served with mayonnaise. In contrast, the high-carbohydrate meal had the lowest energy content, averaging 440.9 ± 30.5 kcal. The high-protein meal yielded a total energy content of 725.7 ± 26.2 kcal.

Due to the drop out of one participant and one study day being repeated, 232 MRI-scans were included in this study. Gastric content volume could be identified and outlined in all MRI-scans for volume determination. Due to incomplete imaging of the stomach at t0, one volume measurement had to be performed in the coronal plane of the TRUFI sequence instead of the axial plane.

**Fig. 2:**
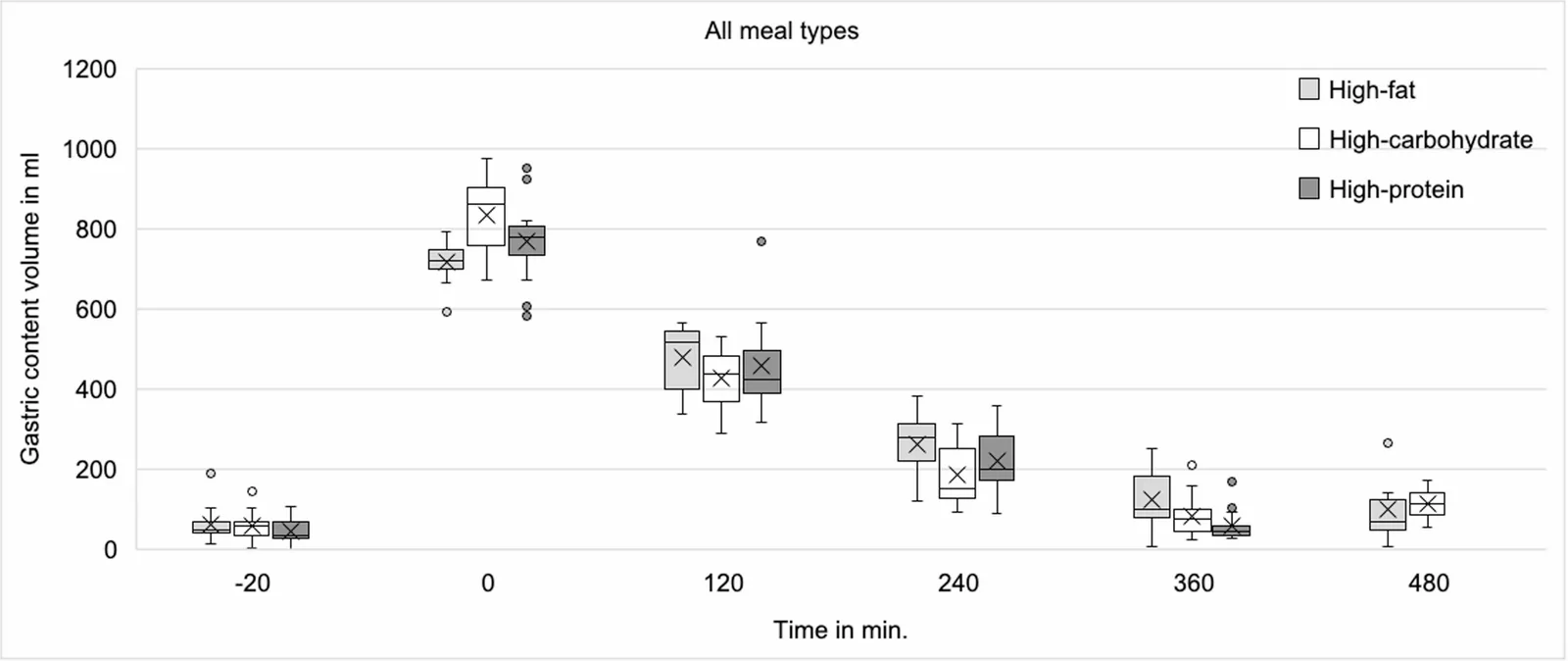
Box plots showing the gastric content volume over time for each standard meal. Boxes show the inclusive median (solid line) and the interquartile range. The mean value is indicated by a cross. Whiskers indicate the 1.5 interquartile range. Outliers are represented as dots. X-axis: time in min, y-axis: gastric content volume in ml

**Fig. 3:**
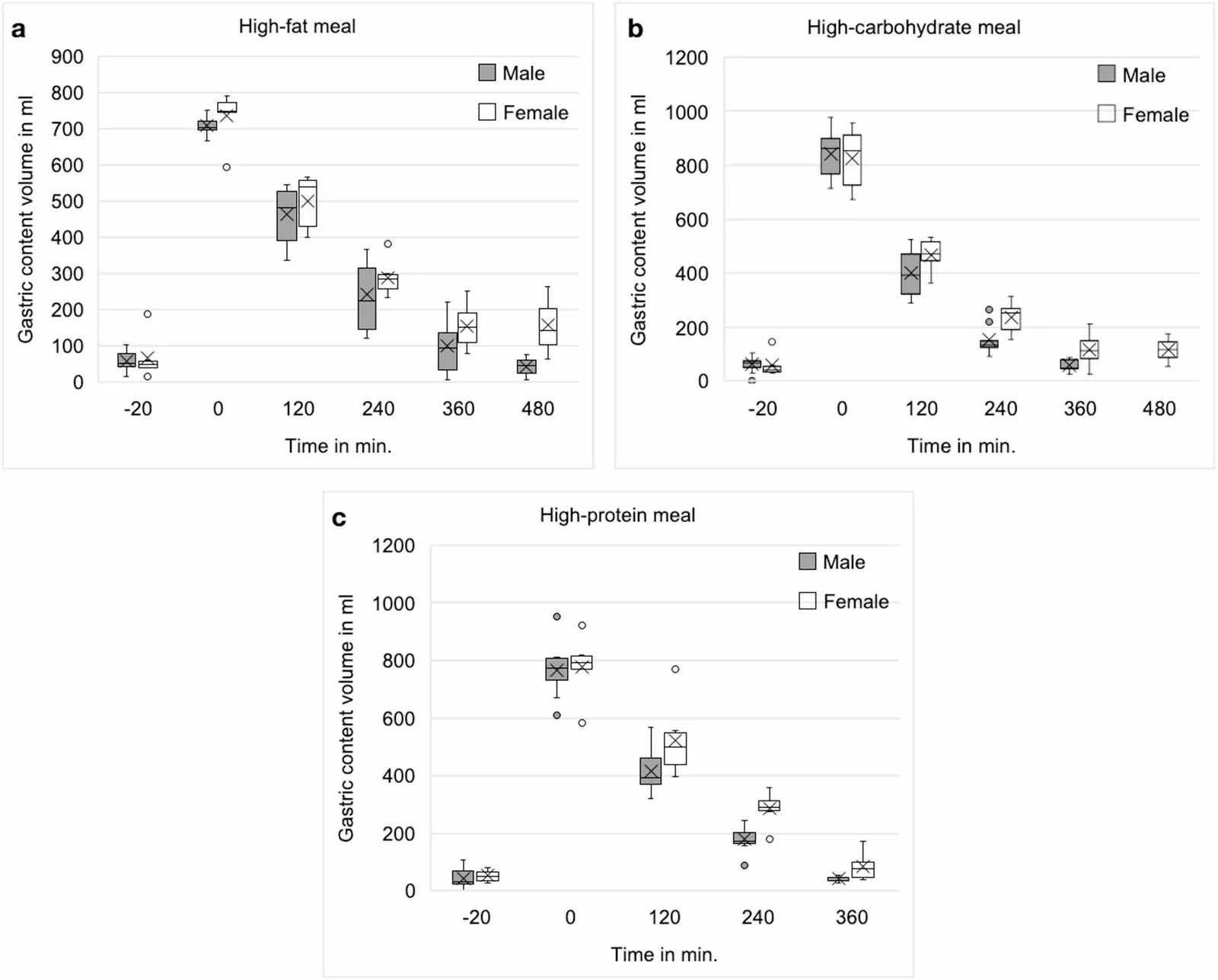
Box plots showing the gastric content volume over time for male and female participants for each meal type (**a**-**c**). Boxes show the inclusive median (solid line) and the interquartile range. The mean value is indicated by a cross. Whiskers indicate the 1.5 interquartile range. Outliers are represented as dots. X-axis: time in min, y-axis: gastric content volume in ml

Figure 2 shows the gastric emptying for the 3 different meal types over time and Fig. 3 for each meal type separated for sex

Across all meal types, most participants showed total gastric emptying after 6 h (36 of 45 consumed meals). Only one male participant reached the defined threshold for total gastric emptying 4 h after consumption of the high-carbohydrate meal.

In the other 9 cases, total gastric emptying was not achieved within 6 h. 5 of these cases reached total gastric emptying after 8 h (1 high-carbohydrate meal, 4 high-fat meals). In the remaining 4 cases (2 female participants), the study day was terminated early due to signs of physical discomfort and hunger (like headaches and vertigo), although the predefined threshold for complete gastric emptying was not reached. 3 of these early terminations occurred in the same participant whose study day had to be terminated early after each meal, with 18–21% of the initial gastric content volume remaining (15–60 ml above the defined threshold; after 6 h for the high-protein meal and after 8 h for the high-fat and high-carbohydrate meals). The gastric content volume of the other participant even increased between the 6-hour measurement and 8-hour measurement (high-fat meal) due to the consumption of water. The detailed drinking protocol is provided in the Supplementary Table 1. 

One male participant had to repeat one study day due to the onset of a migraine during the study which resulted in an early termination of the study day. The participant did not report symptoms until 6 h postprandial, so MRI-scans were performed until early termination. The participant showed delayed gastric emptying and after 6 h gastric content volume increased again from 39.1% to 60.2% of the initial volume directly after meal consumption. In this period, the participant only consumed water, as it was allowed. These results are not included in Figs. 2 and 3.

In general, there was a significantly faster gastric emptying of the high-carbohydrate meal (t₅₀ 132.35 ± 33.38 min) than of the high-fat meal (t₅₀ 187.84 ± 41.17; *p* < 0.001) and of the high-protein meal (t₅₀ 157.57 ± 37.12; *p* = 0.010). There was no statistically significant difference between the gastric half-emptying times of the high-fat and the high-protein meal (*p* = 0.129).

While female participants showed - on average and across all meal types - slower gastric emptying than male participants (Table 5), the differences of the mean t₅₀-times between men and women, only reached significance for the high-protein meal (*p* = 0.003).

**Table 5:** Gastric half-emptying times (t₅₀) of the standard meals

t₅₀ in min			
Different groups	High-fat meal	High-carbohydrate meal	High-protein meal
Total (*n* = 15)	187.84 ± 41.17	132.35 ± 33.38^*^	157.57 ± 37.12
Sex			
Female (*n* = 6)	194.83 ± 32.86	152.18 ± 37.72	188.87 ± 37.52^**^
Male (*n* = 9)	183.18 ± 47.22	119.13 ± 23.85	136.70 ± 17.52
BMI (kg/m²)			
< 20 (*n* = 2)	223.5 ± 38.98	176.82 ± 22.04	180.07 ± 31.67
> 25 (*n* = 2)	187.21 ± 61.79	112.21 ± 20.00	120.78 ± 8.08

All values as means ± standard deviation; t₅₀ (gastric half emptying time)

* significantly faster gastric emptying of the high-carbohydrate meal compared to the high-fat and the high-protein meal (*p* < 0.05); ** female participants showed statistically significant slower gastric emptying than male participants only for the high-protein meal (*p* < 0.017); significance levels are Bonferroni corrected

Participants with particularly slow or fast gastric emptying did not necessarily show a trend across the different meal types, which indicates non-systematic reasons for different gastric emptying times.

The interaction between meal type and sex (meal type*sex) was statistically not significant (*p* = 0.154), indicating that the effect of the meal type on gastric emptying was similar for both female and male.

No underweight participant (BMI < 18.5 kg/m²) was included in this study, but 2 participants had BMIs < 20 kg/m² (one female and one male). They showed slower gastric emptying compared to the average, while overweight participants (BMI > 25 kg/m², *n* = 2, one female and one male) showed faster gastric emptying (Table 5). However, because of the low case number, these findings are only described case-specifically.

## Discussion

This exploratory study demonstrates the effect of different meal compositions commonly consumed on gastric emptying times of healthy persons in solid mixed meals. The main results can be summarized as follows: First, the macronutrient composition of a mixed meal significantly influenced the gastric emptying time. Second, on average, female participants showed slower gastric emptying than male participants, however, this difference was only significant with the high-protein meal. Third, total gastric emptying of a commonly consumed meal mostly occurred after 6 to 8 h across all meal types.

Gastric emptying of the high-carbohydrate meal was significantly faster compared to the high-fat and the high-protein meals, which is consistent with current literature [10]. This may be due to the composition of the meal. Fat and amino acids stimulate the secretion of cholecystokinin, which slows the gastric emptying by “relaxing the proximal stomach, contracting the pylorus, or both” [18].

Although no significant difference in gastric half-emptying times was found between the high-protein and the high-fat meal, 14 participants showed complete gastric emptying 6 h after consuming the high-protein meal, whereas only 9 participants did so after the high-fat meal. Both meals contained a high percentage of total calorie count derived from fat (51.5% for the high-fat meal and 58.9% for the high protein meal). However, the total fat content was 20 g higher in the high-fat meal, resulting in a higher total calorie intake compared to the high-protein meal. This may explain the overall slower gastric emptying, as previous studies have demonstrated that increases in total fat content [19] as well as the overall energy intake can delay gastric emptying [20]. It has to be noted that this finding is only an observation, and no statistically difference was found.

Even though the high-carbohydrate meal had the greatest weight and volume after meal intake, it emptied the fastest compared to the other meals. This contrasts with previous findings suggesting that a general increase in meal volume and weight (regardless of composition) is linked to a longer gastric half-emptying time [21, 22]. However, in these studies, greater meal weight was also associated with greater total calorie content.

In our study, after consuming the high-fat meal, which was the smallest meal in terms of weight and volume after meal intake, with a total calorie count of 1210 to 1340 kcal, gastric half-emptying time was reached after 187.84 min. By comparison, gastric half-emptying time was 132.35 min for the high-carbohydrate meal (440 kcal) and 157.57 min. for the high-protein meal (730 kcal).

These findings may indicate that within the tested meals, factors like meal composition (especially the total fat count) and the total calorie count may be more decisive in delaying gastric emptying times than meal volume and weight.

Complete gastric emptying in healthy subjects mostly occurred between 6 and 8 h across all meal types. This is slower compared to the current literature, in which a ‘normal’ sized meal typically empties within 3 to 5 h, and corresponds to the gastric emptying time of a large meal ranging from 5 to 8 h [10]. However, the authors did not define “normal size”, which complicates a direct comparison. In this study, meal portions procured from restaurants were referenced as ‘normal’ or ‘typical’ meals. One explanation may be bigger portion sizes in restaurants or the increase in portion sizes in the last decades [23].

On average, women showed slower gastric emptying than men, but these differences were only significant in the high-protein meals. These findings are consistent to those of Vasavid et al., who reported significant longer gastric half-emptying times for solid meals in women compared to men using gastric emptying scintigraphy [24]. Further analyses showed that women in the luteal phase of their menstrual cycle as well as women on contraceptive medication had significantly prolonged gastric half-emptying times compared to women in the follicular phase, which may be explained by the influence of sex hormones [25]. Contrary to these findings, Horowitz et al. did not find any statistically significant differences in gastric emptying in a normal menstrual cycle [26]. Madsen also did not find any differences between male and female gastric emptying times [27]. However, this study only included women in their follicular phase.

In our study we did not collect data on the menstrual cycle of the female participants. This is a limitation of this study, as the menstrual phase could mask any differences between the sexes. Because of these heterogenous findings in the literature, when estimating the time of death, it should be considered that women may show slower gastric emptying. However, further studies are needed to investigate the effect of gender on gastric emptying.

The effect of BMI on gastric emptying also remains controversial. In a study of 90 participants, Hellmig et al. could not find a significant correlation between gastric emptying times (determined by ^13^C breath test) and BMI for solid meals [28], while Maddox et al. found a delayed gastric emptying in obese participants using gastric emptying scintigraphy [29]. Due to the small sample size in our study, no statistical analysis of BMI could be performed. However, our descriptive findings are in accordance with Wright et al., who found an accelerated gastric emptying of solid meals in obese participants in a scintigraphic study [30].

Participants in the present study were allowed to stand, sit, or walk to mimic the movement of everyday life. A meta-analysis of 20 studies found accelerated gastric emptying for low-intensity exercises like walking [31]. However, the included studies mostly investigated the effect of liquid meals, so its applicability to solid meals remains uncertain. Nevertheless, when applying gastric emptying times to forensic cases, factors such as physical activity as well as body positioning (e.g. lying supine while sleeping) should be considered, as it may influence the gastric emptying of a solid meal when consumed with a nutritive liquid [32, 33]. However, gastric emptying of a solid meal consumed without a nutritive liquid does not seem to be influenced by posture [22, 34].

MRI has been validated as a method for measuring gastric emptying of a mixed solid/liquid meal [13]. Unlike the gold standard scintigraphy, MRI does not expose individuals to ionizing radiation and is more robust than ultrasonography in suboptimal conditions such as obesity or the presence of intragastric air [35].

Furthermore gastric content volume may appear larger than the ingested meal volume due to secretion [36]. This was also demonstrated in an MRI study by Goetze et al. [37], using liquid test meals.

In contrast to methods such as scintigraphy or breath tests, which only assess the gastric emptying of the labeled meal, MRI measures the total gastric content volume, including secretions [38–40]. Since in autopsies the total gastric content volume is also determined (including salivary and gastric secretions) [12], MRI-based volume measurement seems advantageous in forensic research investigating gastric emptying times.

In this study, participants were allowed to drink water ad libitum, to mimic real life behavior and to improve comfort. In addition, fluid consumption with and after meals is common in daily practice. Fisher et al. demonstrated in a scintigraphic study that the emptying of a solid meal is not influenced by water [41].

However, the measurement points may still have been influenced by water consumption shortly before the MRI scan, potentially resulting in an overestimation of volume. This could particularly explain the noted increase in volume at the 8-hour mark. The effect of liquids other than water on gastric emptying of the solid meals were not investigated. Particularly the consumption of alcohol (ethanol) may delay gastric emptying of a solid meal [42] and should be considered when found in autopsies.

As most of the studies on gastric emptying times, the present study only included healthy participants with no history of medical conditions that might influence gastric emptying.

However, several non-meal-related factors that influence gastric emptying were not accounted for in this study.

One participant showed a delayed gastric emptying while suffering from migraine symptoms. Although anecdotal, this observation is consistent with previous findings in which migraine slowed gastric emptying and may contributed to migraine associated symptoms such as nausea or vomiting [43]. This observation emphasizes that personal factors play a significant role in gastric emptying and must be considered when estimating the time since death. Many factors have been reported to alter gastric emptying such as gastric surgery, medication use or diabetes [11]. Therefore, for a reasonable estimation of time since death, it is necessary to investigate for potential factors that might have altered gastric emptying e.g. in medical records or in autopsy findings [12].

Stomach content based death time estimation should only be used alongside other methods to establish a possible timeframe of death or as corroborative evidence [2, 11, 12]. As with other methods for death time estimation [44, 45], gastric emptying cannot be used to determine an exact time, but rather an interval of several hours [46]. Naturally, this approach requires that the time of the last food intake is known.

## Limitations

This exploratory study only included a small number of participants, all of whom were young and healthy.

Furthermore, the influence of BMI on gastric emptying could not be investigated properly due to mostly normal weight participants.

Although procured from the same restaurants, the composition of the meals varied slightly.

Moreover, only the gastric content volume was assessed by MRI, and not intestinal filling, too, as suggested by Tröger et al. [10], as reliable assessment of intestinal filling by MRI was not possible.

## Conclusion

Gastric emptying is influenced by numerous meal-related and personal factors. Considering all these factors in a forensic case is nearly impossible. Nevertheless, detailed gastric emptying times in healthy persons for commonly consumed meals are presented here to contribute to forensic death time estimation. This approach may seem more practically relevant than considering meal-related factors in isolation.

If the composition of the meal differs from the reference meals, it might be roughly useful to choose a reference meal with a comparable total calorie count as this may be a decisive factor for gastric emptying.

Further validation across larger and more heterogeneous populations is required to improve forensic applicability.

## Supplementary Information

Below is the link to the electronic supplementary material.

Supplementary Material 1 (DOCX 768 KB)

## Data Availability

The datasets generated during and/or analyzed during the current study are available from the corresponding author on reasonable request.
