# Supplementary material for: Gastric emptying times of high-fat, high-carbohydrate and high-protein standard meals using MRI examinations – a contribution to estimating the time since death
Source: Int J Legal Med. 2026 May 19;140(5):3415–25. doi: 10.1007/s00414-026-03836-8 (PMC13499749; doi:10.1007/s00414-026-03836-8)
Supplement: Supplementary file 1 [file 414_2026_3836_MOESM1_ESM.docx]

**Gastric emptying times of high-fat, high-carbohydrate and high-protein standard meals using MRI examinations**

**– a contribution to estimating the time since death**

Dena Farivar^1^, Birte Hellwig^2^, Anna Holzer^1^, Marcel Alexander Drews^3^, Benedikt Michael Schaarschmidt^3,4^, Benno Hartung^1,4^

^1^ Institute of Legal Medicine, University Hospital Essen, Essen, Germany

^2^ Institute for Medical Informatics, Biometry and Epidemiology, University Hospital Essen, Essen, Germany

^3^ Institute for Diagnostic and Interventional Radiology and Neuroradiology, University Hospital Essen, Essen, Germany

^4^ Both authors contributed equally

**Corresponding author:**

Benno Hartung

Institute of Legal Medicine, University Hospital Essen

Hufelandstraße 55, 45147 Essen, Germany

Benno.Hartung@uk-essen.de

**Supplementary Information**

[**Supplementary Table 1** Drinking protocol for each study day and subject](#_Toc225675859)

**Supplementary Table 1** Drinking protocol for each study day and subject
